# Remote assessment and management of patients with dizziness: development, validation, and feasibility of a gamified vestibular rehabilitation therapy platform

**DOI:** 10.3389/fneur.2024.1367582

**Published:** 2024-05-30

**Authors:** Courtney D. Hall, Sheryl Flynn, Richard A. Clendaniel, Dale C. Roberts, Kara D. Stressman, William Pu, David Mershon, Michael C. Schubert

**Affiliations:** ^1^Mountain Home Hearing and Balance Research Program, James H. Quillen Veterans Affairs Medical Center, Mountain Home, TN, United States; ^2^Physical Therapy Program, College of Clinical and Rehabilitative Health Sciences, East Tennessee State University, Johnson City, TN, United States; ^3^Blue Marble Health, Altadena, CA, United States; ^4^Department of Orthopedic Surgery, Physical Therapy Division, Duke University Medical Center, Durham, NC, United States; ^5^Department of Head and Neck Surgery and Communication Sciences, Duke University Medical Center, Durham, NC, United States; ^6^Department of Neurology, Johns Hopkins University, Baltimore, MD, United States; ^7^Department of Otolaryngology Head and Neck Surgery, Johns Hopkins University, Baltimore, MD, United States

**Keywords:** remote assessment, remote patient monitoring, dizziness, vestibular rehabilitation, digital health, usability, feasibility

## Abstract

**Introduction:**

Dizziness is a growing public health concern with as many as 95 million adults in Europe and the United States experiencing vestibular hypofunction, which is associated with reduced quality of life, poorer health, and falls. Vestibular rehabilitation therapy (VRT) is effective in reducing symptoms and improving balance; however, limited access to qualified clinicians and poor patient adherence impedes optimal delivery. The goal of this study was to develop and evaluate the feasibility of a remote therapeutic monitoring VRT Platform application (APP) for the assessment and treatment of vestibular dysfunction.

**Methods:**

User-centered iterative design process was used to gather and integrate the needs of users (clinicians and patients) into the design at each stage of development. Commonly used vestibular patient-reported outcome measures (PROs) were integrated into the APP and adults with chronic dizziness were enrolled to evaluate validity and reliability of the APP compared to standard clinical measures (CLIN). Gaze stabilization exercises were gamified to provide an engaging experience and an off-the-shelf sensor captured eye and head movement to provide feedback on accuracy of performance. A prospective, pilot study design with pre-and post-treatment assessment assessed feasibility of the APP compared to standard VRT (CLIN).

**Results:**

Participants with dizziness wanted a summary rehabilitation report shared with their clinicians, felt that an app could help with accountability, and believed that a gaming format might help with exercise adherence. Clinicians felt that the app should include features to record and track eye and head movement, monitor symptoms, score accuracy of task performance, and measure adherence. Validity and reliability of the digital PROs (APP) were compared to scores from CLIN across two sessions and found to have good validity, good to excellent test-retest reliability, and excellent usability (≥88%ile). The pilot study demonstrated feasibility for use of the APP compared to CLIN for treatment of vestibular hypofunction. The mean standard system usability score of the APP was 82.5 indicating excellent usability.

**Discussion:**

Both adult patients with chronic dizziness and VRT clinicians were receptive to the use of technology for VRT. The HiM-V APP is a feasible alternative to clinical management of adults with chronic peripheral vestibular hypofunction.

## 1 Introduction

Dizziness is a growing public health concern with an estimated 53–95 million adults in Europe and the United States experiencing vestibular hypofunction (1). Vestibular deficits increase with age with one-third of those over the age of 79 demonstrating unilateral and bilateral vestibular hypofunction compared to 2.4% in adults younger than 48 years (1). This is especially concerning because vestibular hypofunction is associated with reduced quality of life, poorer health, and falls which may lead to injury, fear of falling, anxiety and depression (1–3). There is strong evidence in support of vestibular rehabilitation therapy (VRT) to treat dizziness related to peripheral vestibular disorders (4) and emerging evidence in support of VRT for dizziness related to central vestibular disorders (5, 6). VRT is effective for all age groups, including older adults (4). VRT is an exercise-based approach and a critical feature is a home exercise program (HEP) that includes gaze stabilization exercises. Gaze stabilization exercises consist of precise head and eye movements that result in an attenuation of dizziness, improved postural stability, and reduced fall risk. Gaze stabilization exercises should be completed 12–40 min/day for 4–9 weeks depending on severity of vestibular hypofunction and symptoms (4).

While VRT is effective in reducing symptoms and improving postural stability, there are numerous limitations in the current healthcare delivery model, including (1) insufficient access, (2) modest adherence, (3) poor exercise performance by patients, and (4) absence of remote monitoring capabilities. Estimates suggest that VRT is offered to a fraction of individuals in need. The majority of persons seeking medical treatment for dizziness and/or imbalance are initially treated by primary care physicians (55%-80%), often are seen by multiple providers, and frequently do not receive an appropriate diagnosis (7). A study of older adults with dizziness reported that 85% of those seeking medical care saw a general practitioner and 36% saw at least three providers (8). Furthermore, treatment for dizziness or balance problems was prescribed for only 24% of these older patients with medication being the most common treatment prescribed followed by physical therapy (8). As a result, patients with dizziness may not be referred appropriately for VRT. Potential reasons for low rates of referral for VRT are that primary care physicians lack training and knowledge about VRT (9) and/or there is a lack of VRT-trained therapists. Adherence to a HEP is critical to rehabilitation outcomes; yet, studies report low patient adherence, especially over the long-term (10). The factor most strongly associated with reduced adherence is lack of interest suggesting that barriers play a greater role in determining exercise adherence than motivators. Hall et al. reported adherence to a HEP for patients referred for VRT is modest at best (60% compliance) with some participants complaining that the prescribed exercises were monotonous (11). The updated clinical practice guidelines (CPG) for VRT strongly recommends supervised vestibular physical therapy to promote adherence to a HEP; although, the CPG was intentionally vague as to the type of supervision which could include remote therapeutic monitoring or telehealth [(4); https://www.neuropt.org/practice-resources/anpt-clinical-practice-guidelines/vestibular-hypofunction-cpg]. Adequate head speed and accurate eye movement is directly linked to rehabilitation outcomes (12); yet, other than observing head speed and accuracy of eye movement in the clinic, clinicians have no means of offering patients feedback on the accuracy of exercises performed at home. Currently there are no commercially available home programs with remote monitoring capabilities that provide clinicians with head speed and accuracy of eye movement performance data that can be used to correct improper performance.

Together these findings suggest that novel methods are needed to foster adherence to prescribed HEP that are performed at recommended intensity and dose and accurately. The goal of this study was to develop and evaluate Health in Motion-Vestibular (HiM-V), a remote therapeutic monitoring VRT Platform application (APP) for the assessment and treatment of vestibular/balance impairments in adults with complaints of dizziness taking into consideration best practices as outlined in evidence-based clinical practice guidelines (4).

## 2 Methods and materials

### 2.1 Needs assessment

User-centered iterative design process was used to gather and integrate the needs of the users (clinicians and patients) into the design at each stage of development. Semi-structured interviews were performed in adults with chronic dizziness (*n* = 14) and experienced vestibular clinicians and researchers (*n* = 11) to identify desired features, specific user requirements and objectives, and receive feedback from both the patient and clinician perspective. In our previous work, *n* = 10 subjects were required to reach saturation. Additional subjects were recruited until saturation of themes was reached. These interviews focused on discovering how using an app for VRT would be most helpful to the clinicians and beneficial for patients. Product development was guided by this understanding of both patient and clinician preferences and how they would use the APP. This study was approved by the East Tennessee State University/James H. Quillen VA Medical Center Institutional Review Board (IRB #0417.3s). All participants provided written informed consent to participate in the study.

Participants with chronic (>3 months) dizziness were queried about their current use of and comfort with technology, experience with vestibular rehabilitation, performance of a home exercise program, preferred requirements for a digital VRT solution, and feedback on HiM-V. Experienced clinicians were queried about their use of clinical assessments tools of vestibular function, balance and gait, and patient reported outcome measures (PROs), and vestibular exercises to determine essential items to be included in the Platform. Additionally, the clinicians were queried about their use of measures of adherence, telemedicine technology, desired data output from a performance analytics tool, how a VRT app might be used clinically, and a feasible price point.

The interviewer (CDH) took written notes during the interviews and then transcribed these notes into a digital form. Participant data was imported into a computer-assisted qualitative data analysis software (CAQDAS) for qualitative data analysis and one coder coded all qualitative data. Concept codes were pulled from the interview data. Structurally coded data were examined for initial codes, then analyzed, and grouped together to create a more comprehensive picture of the participants' perspectives on using an app to support VRT. Coded data from participants with chronic dizziness addressed four primary categories: (1) technology use, (2) experience with vestibular rehabilitation, (3) experience with a home exercise program, and (4) feedback on the HiM-V prototype following use. Coded data from vestibular clinicians addressed three primary categories: (1) usefulness for a clinician, (2) screening for vestibular dysfunction, and (3) features for a VRT app.

### 2.2 Development of the VRT app, Health in Motion-Vestibular

#### 2.2.1 Integration of patient-reported outcome measures

##### 2.2.1.1 Participants

Sixteen adults over the age of 50 with chronic dizziness were enrolled to evaluate validity and reliability of the patient-reported outcome measures (with the exception of the modified Motion Sensitivity Test, mMST) and usability of the APP. To evaluate mMST a separate group of 10 healthy adults over the age of 18 years with chronic motion-provoked dizziness were enrolled and five Doctor of Physical Therapy students served as test administrators and evaluated usability of the digital version of mMST. This study was approved by the East Tennessee State University/James H. Quillen VA Medical Center Institutional Review Board (IRB #0417.3s) and the VA Central Institutional Review Board (Protocol #1717092). All participants provided written informed consent to participate in the study.

##### 2.2.1.2 Protocol

To establish concurrent validity participants completed the assessments under standard clinical practice conditions (CLIN), and by using the HiM-V in-app assessments (APP). The CLIN assessments involved standard verbal instruction, a stopwatch, a metronome and paper-based questionnaires. The APP instructions mirrored the CLIN instructions as closely as possible. The APP instructions differed if the paper-based questionnaires referenced “marking an X,” which was translated to a digital format using “select a response” instructions, for example. The APP tests also limited the number of questions per screen to 1–2 questions, compared with the CLIN test, which could contain many more questions on a single sheet of paper. The APP tests required a response for each question, whereas a question could be skipped in the CLIN version. Scores for each assessment were calculated and displayed on the screen immediately upon completion of the assessments for the APP version; whereas the CLIN version required the test administrator to manually score each test. To establish test-retest reliability participants returned within 2 days to 2 weeks to retake the assessments. The order of the assessments was counterbalanced across participants with half beginning with CLIN and half beginning with APP and then the order was reversed for the second session. To examine usability, participants rated HiM-V using the System Usability Scale [SUS; (13)]. Permissions and/or licenses were obtained as needed to integrate commonly used PROs for vestibular assessments into the app including the modified Activities-specific Balance Confidence Scale, Dizziness Handicap Inventory, Disability Rating Scale, Visual Analog Scales to rate Dizziness Interference in Activities and intensity of Oscillopsia, Disequilibrium, and Dizziness, and mMST.

##### 2.2.1.3 Patient-reported outcome measures

The Activity-specific Balance Confidence scale (ABC) is a self-report measure of confidence in balance ability while performing 16 different activities such as walking on level surface, negotiating stairs and slopes, and slippery surfaces (14). A modified version (mABC) was utilized in which participants rated their confidence for each item on a 5-point rating scale (0% [no confidence], 25%, 50% [moderately confident], and 75%, 100% [completely confident]) compared to the standard rating scale (15). An overall average was calculated, and a higher score indicates greater confidence in balance ability.

The Dizziness Handicap Inventory (DHI) is a 25-item self-report measure of the impact of dizziness or unsteadiness on daily activities and is the most commonly used patient-reported outcome measure of dizziness (16, 17). Responses to whether specific activities or situations increase the problem include yes, no and sometimes. The DHI assesses self-perceived handicap, and total scores can range from 0 to 100 with higher scores indicating greater perceived handicap due to dizziness or imbalance. The minimal detectable change (MDC) for DHI total score is 18 points which can be used to evaluate therapeutic change and responsiveness to therapy (16).

The Disability Rating Scale (DRS) is a global self-report of disability on a 6-point scale from 0 to 5 with 0 indicating no disability and 5 indicating long-term severe disability (18). The DRS has excellent test-retest reliability with an MDC of a 1-point change in score (19). The DRS has been shown to predict rehabilitation outcome in individuals with vestibular dysfunction such that patients with higher perceived disability (scores of 4 or 5) were less likely to improve with vestibular rehabilitation (18).

**Figure 1:**
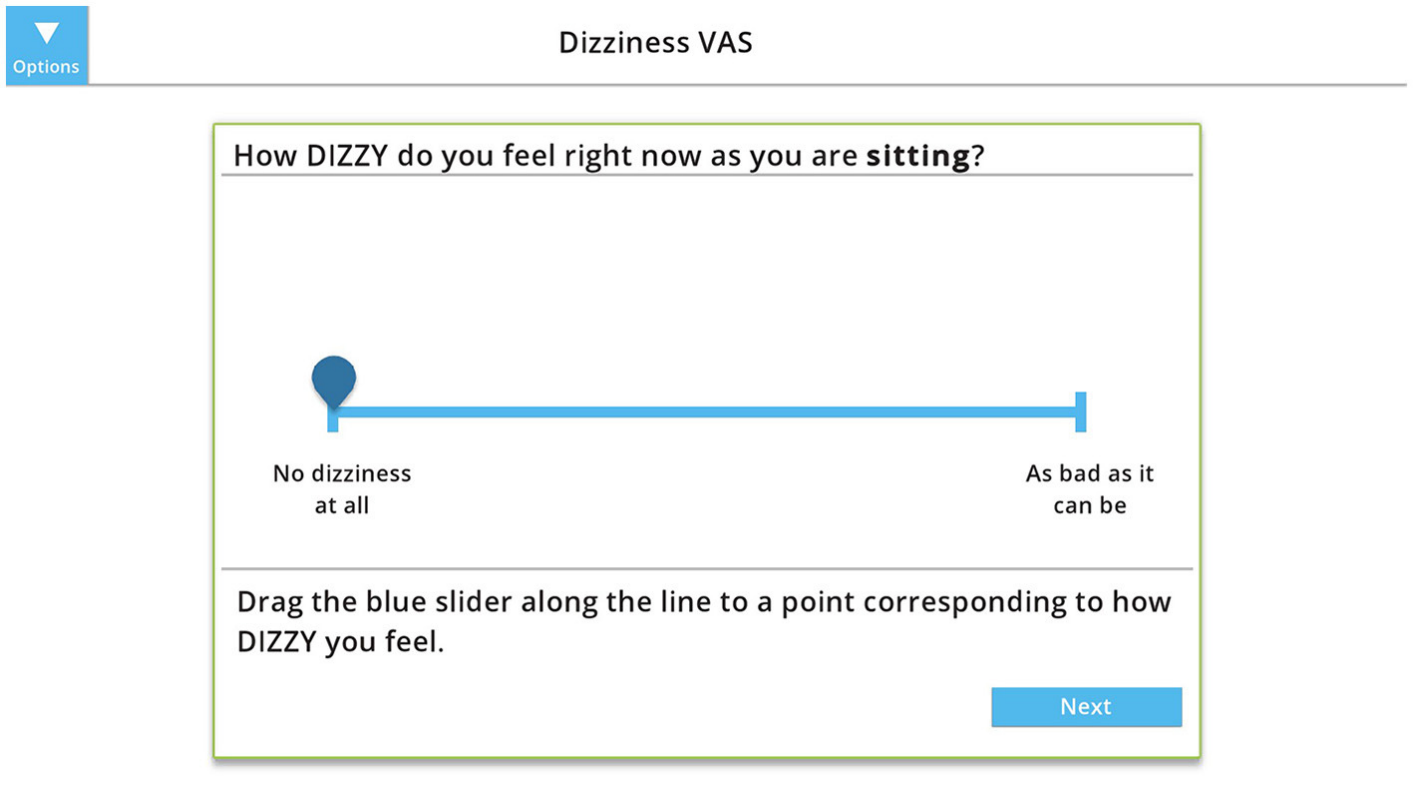
Example of the digital visual analog scale (VAS) to measures intensity of dizziness at baseline (while sitting). The participant slides the marker or taps the line to indicate their response, which is measure from 0 (“no dizziness at all”) to 10 (“as bad as it can be”). Copyright (2024) Blue Marble Health. Reprinted with permission.

Visual Analog Scales (VAS) are commonly used to assess intensity of symptoms using a 10-cm line with word cues anchored at either end to represent the extremes of symptoms—“no symptoms” at one end and “as bad as it can be” at the other end. To assess the percentage of time dizziness interferes with activities (Dizziness Interferes), patients mark on a 10-cm horizontal line and the distance from 0 to the mark is measured (19). VAS for Oscillopsia, Disequilibrium and Dizziness involved rating symptoms on a 10-cm vertical line at baseline (while sitting) and then after walking (for Oscillopsia or Disequilibrium) or rotating the head for 1 min (for Dizziness) (19, 20). The Dizziness VAS has modest reliability with the MDC being a change of 4.3 cm (19). The score is the distance from 0 to the mark. For the digital VAS, the participant slides a marker or taps the line to indicate their response (Figure 1).

The mMST evaluates motion provoked dizziness through performance of ten different movements such as head turns, 360° turn, and trunk bending in a standing position (21). A clinician records each participant's verbally reported dizziness intensity using a 10-point scale (0 indicates no dizziness at all and 10 is the worst symptoms possible) at baseline and directly following each movement. A total score, the Motion Sensitivity Quotient (MSQ), is calculated based on change in symptom intensity and duration of symptoms following each movement.

##### 2.2.1.4 Data analysis

Data were summarized using descriptive statistics. To evaluate concurrent validity, bivariate Pearson's correlations were calculated. The correlation coefficient values were interpreted as follows: strong relationship (≥0.75), moderate to good (0.50–0.75), low to fair (0.25–0.50), and little or no relationship (≤ 0.25) (22). To evaluate test-retest reliability, intraclass correlation coefficients (ICCs; two-way random effects model 2) were calculated. Reliability coefficients were interpreted as follows: poor reliability (<0.5), moderate (0.5–0.75), good (>0.75–0.90), and excellent (>0.90) (23). The HiM-V Platform's usability and learnability was measured using SUS and the standard SUS score was calculated and then interpreted using the Sauro–Lewis curved grading scale (13).

#### 2.2.2 Development of gamified gaze stabilization exercises

The main goal for creating a gaming format for the gaze stabilization exercises was to improve HEP adherence by providing an engaging, meaningful, and enjoyable way to perform these exercises. An off-the-shelf sensor for “plug-n-play” ease of use would be incorporated to capture eye and head movement and provide immediate feedback on accuracy of performance. Other objectives for the system included affordability, the ability to track progress over time, enable clinicians to remotely monitor patient performance, symptoms, and adherence, and to make the product commercially available to patients and clinicians. The vestibular game system consisted of a laptop, Health in Motion software, and Tobii Eye and Head Tracker. For the validation of the Tobii gaze and head tracking, PC laptops with screen size of 15.6 inches and 1920 x 1080 resolution were utilized. The Tobii sensor was centered at the base of the laptop screen according to manufacturer instructions and the participant was positioned ~30 inches from the screen.

#### 2.2.3 Validation of Tobii sensor into app

The Tobii 4C sensor was chosen to control the game mechanics because it is an off-the-shelf gaming device that tracks both gaze and head movement, which provides the user with a measure of gaze (i.e., where a subject is looking). This offers a significant advantage to eye trackers alone that do not consider head position and thus cannot accurately measure or report gaze, which requires knowing both eye and head position. The Tobii 4C outputs a stream of timestamped samples with gaze and head position (x, y relative to screen position) which are converted into Euler angles (x, y, z with rotation measured in degrees) in our software. For x, y screen position, the bottom-left of the screen is (0, 0) and the top-right is (1, 1). The x (pitch), y (yaw), and z (roll) Euler angles are at (0, 0, 0), when the user's head is facing straight ahead at the screen. Instantaneous angular head velocity (in yaw and pitch) was computed by finding the difference in head angle divided by the difference in time between two consecutive samples.

The latency between head motion (tracked by Tobii) and graphic display in the HiM-V platform was determined using a head-mounted angular rate sensor (InvenSense, TDK ICM-42688 6-axis digital MEMS IMU) and an analog light sensor, both connected to a small microcontroller (Teensy 3.2, PJRC.com), which stored data from both sensors together. The analog data from the light sensor was sampled by an analog-to-digital converter in the Teensy microcontroller. This system was independent of the Tobii, and its purpose was to directly measure the total latency from head movement to graphic display, so no synchronization with the Tobii was required. The angular rate sensor sample rate was 500 Hz, and the light sensor latency was <1 ms, allowing latency measurement to within 2 ms, a small fraction of the total vestibular game system latency. The analog signal from the light sensor was sampled at the instant that the angular rate sensor sample became available (software latency within the microcontroller is <1 ms). The light sensor was taped to the monitor at the location where the graphic display would appear and detected the display, while the subject moved their head. This allowed a direct measurement of total system latency, which was found to be ~130 ms. The goal of validation was to determine the accuracy of head velocity and gaze position measured by Tobii during game play and used by the HiM-V software.

Validation of Tobii eye and head tracker was completed with five healthy adults and adults with vestibular hypofunction. To determine the accuracy of head tracking data used by HiM-V software, head position in space data from HiM-V and infrared (IR) goggles with angular rate sensors (GN Otometrics NA, Schaumburg, IL) were collected simultaneously while performing gamified VORx1 for 30 s at slow (60°/s) and fast (180°/s) speeds. The data were then analyzed using the linear regression statistical function in Excel. The gamified VORx1 game mechanics consisted of gazing at an on-screen target (a cross) while moving the head at a speed above the threshold speed. The eye position was considered on target when gaze, as measured by Tobii eye and head tracker, was within the radius of the visible target image on the computer screen, as determined by HiM-V. The target image size was calculated based on its pixel size set; that is, a ratio of computer screen height to target size (small, medium, and large; Supplementary Table 5).

To determine head rotation speed and direction, raw Tobii head position data were processed using the HiM-V software. HiM-V grouped instantaneous angular head velocity samples into chains moving in the same direction (e.g., clockwise movement followed by another clockwise movement). The longest chain of samples moving in the same direction was selected and used to estimate the current movement direction (clockwise or counterclockwise). Head rotation speed (°/s) was computed by taking the mean of the absolute values of all the angular velocity samples within these chains. If the head rotation speed estimated by the system dropped below the minimum head speed threshold, then HiM-V would register the head movement as too slow. Only samples within the sample window length of 0.25 s were included in the calculation. When assembling the instantaneous angular head velocity samples into chains, one sample moving in the opposite direction of the chain was permitted due to the inherent noisiness of the Tobii sensor data. At least two angular head velocity samples moving in the same direction were required to form a chain. Eyes remaining on target and head moving above threshold caused a plant to grow larger in size. To determine the accuracy of gaze position data used by HiM-V and the accuracy of game feedback to patients, the following data were collected from HiM-V while playing VORx1 at slow and fast speeds: gaze position (x, y on screen), head position (Euler angle), head velocity (°/s), eyes on target (yes/no), head on target (head velocity faster than target velocity; yes/no), and plant size.

Clinicians requested an integrated digital Dynamic Visual Acuity test (dDVA) to avoid launching two unique software tools when creating the appropriate home program; thus, we designed and developed a dDVA modeled after the NIH toolkit computerized DVA (24). A metronome was used to pace the head movements. The participant was instructed to move the head in one direction (i.e., from left to right or right to left) with the beat of the metronome. The algorithm for dDVA was tested at three different head velocities (60, 90, and 120 bpm). To determine the actual head velocity threshold at which the optotype (letter “E”) flashed, the head-mounted angular rate sensor was synchronized with the analog light sensor pointed toward the computer screen (as described above), which allowed determination of the head velocity and direction when the letter appeared on the screen.

The dDVA protocol began with calibration of the Tobii eye tracker. Participants were prompted to position themselves 30 cm from the laptop screen based on the distance detected by the Tobii head and eye tracker. Integrated instructions and animations were developed and provided before each test to model appropriate behavior. The dDVA testing software included both static (SVA) and dynamic visual acuity (DVA) testing. During both SVA and DVA testing, an “E” optotype was flashed on the screen in different randomized orientations (up, down, left, or right). Optotype size was progressively decreased in size by 0.1 LogMAR until the participant made more than two consecutive incorrect responses at a specific optotype size. Participants input their own answers manually using the laptop's arrow keys (up/down/left/right) that corresponded with the direction the “E” optotype was facing.

### 2.3 Pilot intervention study

The pilot study was approved by the East Tennessee State University/James H. Quillen VA Medical Center (JHQVAMC) Institutional Review Board (IRB #0417.3s) and the Alpha Institutional Review Board as the Central IRB for four additional sites (IRB #2020.1-0201). Additional sites included Department of Otolaryngology Head and Neck Surgery at Johns Hopkins University, Vestibular Disorders Clinic at Duke University, Emory Dizziness and Balance Clinic at Emory Healthcare and Vestibular Therapy Specialists in Bend, Oregon. All participants provided written informed consent to participate in the study.

#### 2.3.1 Design

This was a prospective, exploratory pilot study with pre- and post-treatment assessment.

#### 2.3.2 Participants

Thirty-two adults with vestibular hypofunction were enrolled. Inclusion criteria included diagnosis of vestibular hypofunction based on vestibular function testing (e.g., caloric test, head impulse test) within the past 12 months, documented dizziness and/or imbalance problems, able to stand for at least 3 min and walk without assistance of another person with or without an assistive device, and able to transport to the site. Exclusion criteria included cognitive impairment as measured by the Mini-Mental State Exam (<24/30) or clinical diagnosis, progressive medical issues that would affect mobility (e.g., Parkinson's Disease), significant orthopedic issues that would limit participation in gaze stability exercises (e.g., significantly limited cervical range of motion or pain), or significant visual impairment that would limit the ability to utilize the APP for training.

#### 2.3.3 Protocol

Potential participants were recruited through Physical Therapy, Audiology, Otolaryngology, or Rehabilitation Medicine clinics and were evaluated for inclusion/exclusion criteria by a trained research assistant or physical therapist specializing in vestibular rehabilitation. Participants were randomized into study groups defined by the method that the HEP was provided: standard vestibular rehabilitation therapy (S-VRT) home program or digital vestibular rehabilitation therapy (D-VRT) home program using HiM-V. To better understand user preference, participants were offered the option to switch groups for any of the following reasons: boredom, poor adherence, preference to receive (or avoid) feedback while doing exercises, preference for a gamified (or non-gamified) intervention, preference to share (or not share) data with supervising clinician, insurmountable technical difficulties, or excessive increase in symptoms.

At the initial session, participants were evaluated on self-reported outcome measures, balance and gait measures, and were instructed in a customized HEP for balance and gait impairments. Participants were seen weekly (either face-to-face or via phone call) for balance and gait training and progression of HEP. Participants recorded adherence with exercises on a weekly calendar. After 4 weeks of VRT, all assessments were repeated.

#### 2.3.4 Outcome measures

The patient-reported outcome measures were completed using the HiM-V app and included the modified Activities-specific Balance Confidence scale, Dizziness Handicap Inventory, Disability Rating Scale, and Visual Analog Scales for Dizziness Interference, Oscillopsia, Disequilibrium, and Dizziness. In addition, measures of balance and gait were completed including the Functional Gait Assessment (FGA), modified Clinical Test of Sensory Interaction and Balance (mCTSIB), preferred gait speed and a subset of participants (at JHQVAMC) were assessed using computerized Dynamic Visual Acuity (cDVA; NeuroCom InVision). Additionally, all participants completed a paper exercise log to quantify adherence to their HEP. Adherence (number of exercises completed each week/number of exercises assigned each week) to gaze stabilization exercises and balance and gait exercises were calculated separately. The D-VRT group completed the System Usability Scale during the final session (13).

#### 2.3.5 Intervention

Each participant received a customized VRT HEP per standard care consisting of gaze stabilization exercises, including adaptation and substitution exercises, balance and gait exercises and walking for endurance. Gaze stabilization exercises based on vestibular adaptation involve head movement while maintaining focus on a target, which may be stationary (VORx1) or moving (VORx2). Gaze stabilization exercises based on substitution promote alternative strategies. For example, gaze shifts exercise between targets involves a large eye movement to a target followed by head movement to face the target potentially facilitating compensatory saccades. The remembered target exercise involves focusing on a target, closing the eyes, and turning the head while maintaining the eyes on the (remembered) target.

The gaze stabilization HEP was delivered using standard paper handouts (S-VRT) or game format using HiM-V app (D-VRT). All participants received verbal instructions, demonstration of, and feedback on, accurate performance of the HEP per group assignment. The supervising clinician evaluated the patient's ability to accurately perform the exercises at each clinic visit (1x/week) and provided additional training/feedback as needed. All patients were instructed to complete the gaze stabilization exercises daily (with the option to take 1 day off per week), 5 times per day for 4 weeks.

Both S-VRT and D-VRT were provided with a written balance and gait HEP designed to improve postural stability and mobility with progressively more challenging tasks. Balance exercises included maintaining stability in standing with altered base of support and altered vision and/or somatosensory cues, dynamic weight shifting and dynamic stepping on firm or foam surface. Gait activities included walking with head turns, varied gait patterns (e.g., narrow, backwards, sidestep, and grapevine), and with and without vision. Walking for endurance was included in the HEP. The customized HEP was based on identified impairments and functional limitations and progressed according to ability.

#### 2.3.6 Data analysis

Data were summarized using descriptive statistics. To determine the effectiveness of S-VRT compared to D-VRT, 2 x 2 (Time by Group) mixed ANOVAs were performed for the outcome measures (alpha = 0.05) and significant findings were followed with univariate analyses. To determine group differences in exercise adherence, independent *t*-tests were performed (alpha = 0.05).

## 3 Results

### 3.1 Needs assessment

#### 3.1.1 Participants with chronic dizziness

##### 3.1.1.1 Participants

Sixteen individuals were enrolled in the study and 14 completed the entire protocol, including interviews. All participants were white males over 50 years of age (69.6 ± 5.8 years; Supplementary Table 1) and 13 were veterans. Eleven participants had a diagnosis of vestibular dysfunction and three had normal vestibular function and imbalance; 10 reported a history of at least one fall in the previous year.

##### 3.1.1.2 Technology use

All participants reported regular interaction with some form of technology, most frequently, telephones and computers (Supplementary Table 2). Only two participants did not own a computer. The primary uses of computers were for search engines, email, and shopping. Thirteen reported being comfortable using a computer while nine did not consider themselves to be “tech-savvy.” Eleven were interested in having a summary rehabilitation report shared with their clinicians (MD, PT, OT) automatically. Three recommended reminders as an app feature to help with accountability. Seven participants were interested in a gaming format and 11 believed that a gaming format might help with adherence. Twelve were willing to pay for an app with five participants suggesting they would be willing to pay $50 or less.

##### 3.1.1.3 Vestibular rehabilitation

Six participants had no experience with VRT, six had positive experiences and considered VRT as helpful, useful, or enjoyable; whereas two had negative experiences with VRT with increased symptoms or found it not to be beneficial. Six of eight reported ever having personalized home exercise programs and reported that they were explained thoroughly, and three received demonstrations before being sent home to implement the traditional paper-based home program, none of which included technology. The traditional home programs were not considered easy to adhere to by many of the participants. Participants indicated that forgetting was a barrier to adherence, and that solutions could include reminders and interaction with someone for accountability; additionally, feedback on their progress, an engaging app, and feedback to their clinician would assist with adherence (Supplementary Table 3). Six participants thought that an app might be useful and aid with accountability. Twelve of the 14 participants would consider using an app for VRT.

##### 3.1.1.4 Feedback on app

After using HiM-V, 11 (73%) agreed that the app was easy, simple, straightforward, or self-explanatory. Overall, test instructions were considered adequate. All participants expressed a desire for some form of visual representation of their data with more than half preferring graphs over tables. The artwork received mixed responses with the majority being neutral- either they didn't notice, or it was “elementary but not a deal breaker.” To increase adherence, participants suggested adding additional feedback about their performance, reminders to exercise, and sending results to clinicians as features that would encourage users to continue using the app.

#### 3.1.2 Experienced VRT clinicians

##### 3.1.2.1 Participants

Fourteen experts in vestibular rehabilitation completed phone interviews and 12 provided written consent for their data to be included in research analyses. Experts included nine females (75%) and three males (25%) ranging from 36 to 71 years old (mean = 54.8 ± 10.0 years). Years of physical therapy practice ranged from 12 to 42 years (mean = 29.6 ± 10.5 years) with an average of 20.6 ± 9.5 years in specialty practice. All experts spent at least 25% of their time performing vestibular rehabilitation and half spent more than 75% of their time in clinic.

##### 3.1.2.2 Features for a VRT app

The consensus was the app must include features for: (1) recording and tracking eye and head movement, (2) monitoring symptomatic response to movement, (3) scoring accuracy of task performance, and (4) measuring adherence to the prescribed home exercise program (Supplementary Table 4).

Eleven of the clinician experts stressed the ability to track movement and to compare eye and head movements for accuracy, which is not currently available in rehabilitation tools. The frequency, amplitude, and duration of exercises would be helpful to the clinicians to evaluate patient progress. Ten of the clinicians deemed the summary/weekly reports as highly critical for following patient progress, measuring adherence, and helping the clinician alter the therapy program by showing performance changes.

Five of the clinicians suggested providing patients an option to rate their symptoms before and after exercises to enable patients to compare and relate their symptoms to movement. For the clinician, this could help flag changes and understand better ways to treat the symptoms.

The clinicians suggested that measurement of performance over time could be helpful for a clinician to observe the patient's progress. The clinicians suggested that performance could be displayed as the outcome of an individual PRO or exercise (raw scores) and for overall progress evaluation as graphs and/or tables. The clinicians felt that the performance measures should be available to patients to view their progress and these measures may provide the patient with ownership, motivation, and challenge the patient. The app should record the performance of activities, so the therapist can measure the actual performance of HEP instead of relying on subjective measurements. The clinicians felt that an app may increase a patient's likelihood of adhering to the program by being consistently engaging, challenging patients to be committed and involved in their progress while sharing the data with clinicians.

The clinicians discouraged an app designed for adults with childlike graphics. Furthermore, it was recommended that the app theme be chosen by the patient, as not all people respond positively to similar styles. The clinicians felt that feedback from the app may reinforce patients' involvement with at-home rehabilitation, and the app is an opportunity to give patients autonomy (with guidance) and ownership of their rehabilitation progress.

A word cloud with the top 50 most frequent words was produced from the expert data using CAQDAS (Supplementary Figure 1). Head, movement, exercises, eye, speed, patient, time, and symptoms had the highest frequencies; thus, these words are larger than the rest.

### 3.2 Vestibular rehabilitation therapy app

#### 3.2.1 Integration of patient-reported outcome measures

Fifteen male participants (mean age: 69.7 ± 5.7 years; range: 53–76) with chronic dizziness completed the two testing sessions for all PROs other than the mMST (Table 1). Concurrent validity was measured by comparing outcomes from CLIN and APP and was generally found to have strong correlations with the exception of intensity of symptoms measures by VAS (Table 2). Test-retest reliability was measured by comparing outcomes from the tests across two sessions (mean = 9.7 ± 5.8 days between session 1 and 2) and was found to be good to excellent, with the exception of intensity of symptoms measured by VAS (Table 3). Adults with chronic dizziness were included because their symptoms might fluctuate less than patients with acute dizziness; however, this did not prove to be the case as demonstrated in the reliability coefficients for symptom severity based on visual analog scales (Table 3). The system usability score was 92.5 ± 9.3 for session 1 and 90.2 ± 10.8 for session 2 indicating excellent ease of use or the equivalent of an A+ score (13).

**Table 1:** Characteristics of participants with chronic dizziness (*n* = 15).

**Variable**		
Age (years)		69.7 ± 5.7 (range: 53–76)
Vestibular diagnosis (*n*)	Normal vestibular, imbalance	3 (20%)
	Unilateral peripheral vestibular hypofunction	9 (60%)
	Bilateral peripheral vestibular hypofunction	2 (13%)
	Benign paroxysmal positional vertigo	1 (7%)
Time from onset of symptoms (months)		154.1 ± 226.5 (range: 4–720)
Education	HS or less	4 (26%)
	Vocational training, some college/AA	4 (26%)
	Bachelor's or higher degree	7 (47%)
Assistive device use	None	9 (60%)
	Cane	5 (33%)
	Walker	1 (7%)
Functional Comorbidity Index (*n*)		5.0 ± 2.5 (range: 0–9)
History of any falls	no/yes	4/11
>1 fall (recurrent falls)	no/yes	10/5
Composite Physical Function (/24)		21.3 ± 2.5 (range: 16–24)
Fall Risk Questionnaire (/15)		7.8 ± 3.5 (range: 0–13)
High risk (≥4/15)	no/yes	1/14
Timed up and go (s)		11.3 ± 2.1
Preferred gait speed (m/s)		0.95 ±0.13

Data are presented as n (%) or mean ± SD.

**Table 2:** Mean ± SD for patient-reported outcome measures and correlation coefficients (Pearson's *r*) to evaluate concurrent validity (CLIN vs. APP).

**Test**	**Session 1**			**Session 2**		
	**CLIN**	**APP**	***r***	**CLIN**	**APP**	***r***
Activities-specific Balance Confidence (%)	66.4 ± 19.1	62.7 ± 20.5	0.95	60.0 ± 26.6	59.1 ± 25.6	0.99
Dizziness Handicap Inventory (/100)	36.7 ± 22.6	37.5 ± 21.8	0.97	38.5 ± 22.8	37.9 ± 23.7	0.98
Disability rating scale (*n*)			0.83			0.96
None (0–1)	9	7		7	7	
Mild (2)	1	3		1	0	
Moderate (3)	2	3		5	5	
Severe (4–5)	3	2		2	3	
% of time dizziness interferes (%)	25.0 ± 26.5	24.9 ± 24.8	0.92	38.1 ± 30.9	32.6 ± 25.8	0.84
Oscillopsia VAS (/10)						
Sitting	0.4 ± 0.5	0.4 ± 0.6	−${\text{0.06}}^{\bar{\text{T}}}$	2.1 ± 1.9	1.1 ± 2.2	${\text{0.19}}^{\bar{\text{T}}}$
Walking	1.3 ± 1.5	1.1 ± 2.0	0.62^*^	2.6 ±2.9	2.1 ± 2.8	0.71^**^
Difference	0.9 ± 1.4	0.6 ± 1.8	0.68^**^	0.5 ± 1.4	1.0 ± 2.4	0.82^**^
Disequilibrium VAS (/10)						
Sitting	0.8 ± 1.2	0.9 ± 1.7	${\text{0.50}}^{\bar{\text{T}}}$	1.2 ± 1.9	1.6 ± 2.4	0.73^**^
Walking	3.2 ± 3.2	1.9 ± 2.4	0.65^*^	2.8 ±2.6	2.1 ± 2.7	0.79^**^
Difference	2.4 ± 2.6	1.0 ± 1.3	0.79^**^	1.5 ± 1.5	0.4 ± 2.0	${\text{0.25}}^{\bar{\text{T}}}$
Dizziness VAS (/10)						
Sitting	1.5 ± 1.6	1.2 ± 2.0	0.63^*^	2.0 ± 2.3	1.5 ±2.1	0.92^**^
1-min head turns	4.5 ± 3.2	4.0 ± 2.8	0.74^**^	4.6 ± 3.5	4.0 ±3.2	0.78^**^
Difference	3.0 ± 2.2	2.8 ± 2.1	0.58^*^	2.6 ± 2.3	2.5 ± 2.8	0.75^**^
System Usability Scale (%ile)	—	92.5 ± 9.3		—	90.2 ± 10.8	

Correlations are significant at p <0.001 unless otherwise indicated: ^*^p <0.05; ^**^p <0.01; ${\;}^{\bar{\text{T}}}$ p > 0.05.

**Table 3:** Test-retest reliability of CLIN and APP measures.

**Variable**	**CLIN**		**APP**	
	**ICC**	**95% CI**	**ICC**	**95% CI**
Activities-specific Balance Confidence (%)	0.86	0.62–0.95	0.90	0.74–0.97
Dizziness Handicap Inventory (/100)	0.90	0.73–0.97	0.86	0.64–0.95
Disability rating scale (0–5)	0.86	0.64–0.95	0.68	0.28–0.88
% of time dizziness interferes (%)	0.50	−0.003–0.80	0.75	0.40–0.91
Oscillopsia VAS (/10)				
Sitting	−0.07	−0.55–0.44	−0.10	−0.57–0.42
Walking	0.46	−0.05–0.78	0.59	0.12–0.84
Difference score	0.69	0.29–0.88	0.70	0.30–0.89
Disequilibrium VAS (/10)				
Sitting	0.42	−0.09–0.76	0.56	0.07–0.84
Walking	0.64	0.21–0.86	0.77	0.42–0.92
Difference score	0.47	−0.03–0.78	0.56	0.06–0.83
Dizziness VAS (/10)				
Sitting	0.73	0.36–0.90	0.91	0.74–0.97
1–min head turns	0.76	0.43–0.91	0.77	0.42–0.92
Difference score	0.69	0.30–0.88	0.55	0.05–0.83

Ten adults with chronic motion-provoked sensitivity (mean age = 44.7 ± 15.8 years; range: 20–61 years; eight females) completed the two testing sessions 2 days to 1 week later (mean = 3.8 ± 2.6 days. There was a strong relationship between APP compared to CLIN (1st session: ρ = 0.95, *p* < 0.001; 2nd session: ρ = 0.96; *p* < 0.001). Test-retest reliability of the digital mMST was excellent (ICC = 0.92). The system usability score was 88 ± 11.4 indicating excellent ease of use equivalent to an A+ score (13).

#### 3.2.2 Gamified gaze stabilization exercises

**Figure 2:**
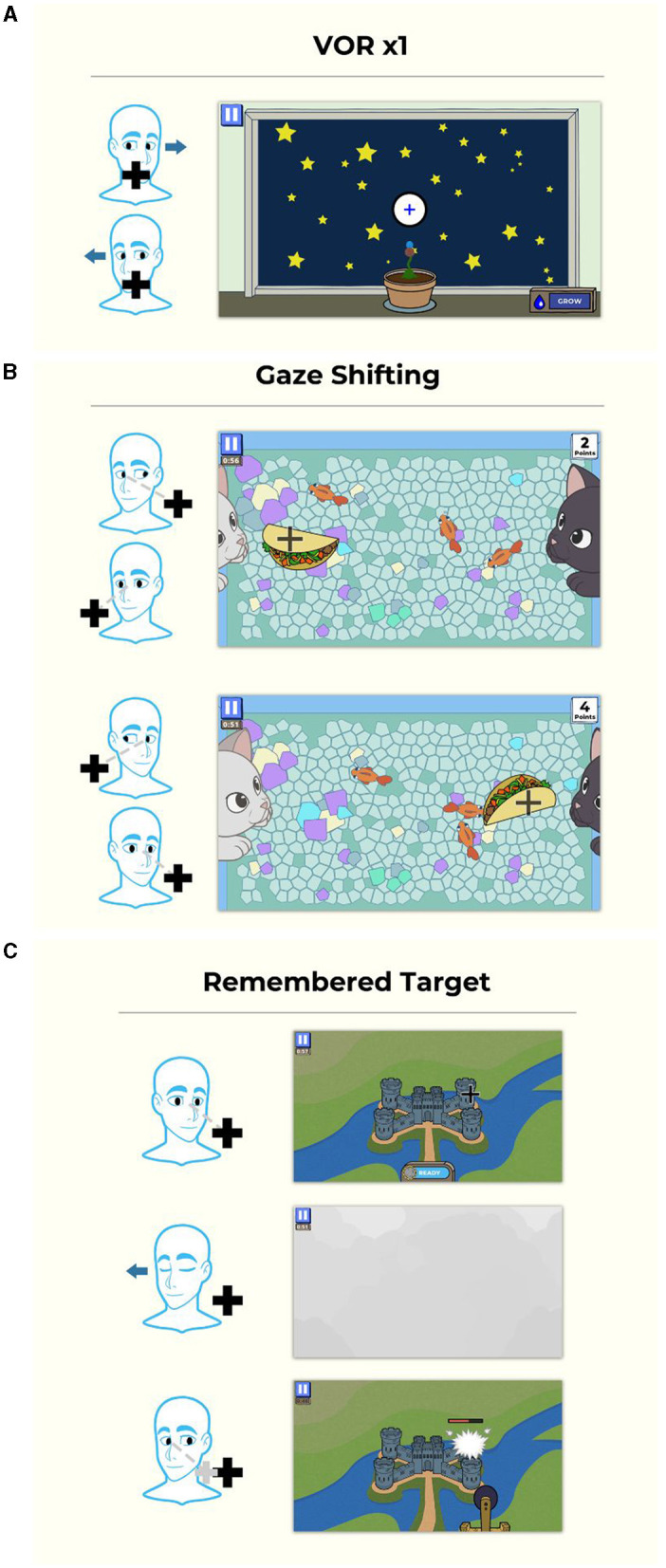
Gamified format for gaze stabilization exercises. **(A)** VORx1 and VORx2 (“Gaze Garden”) use a garden format in which the patient selects a plant to grow, then they “grow” it by performing accurate eye and head movement at a selected head velocity, and after the game, plant it in the garden. **(B)** Gaze shifting exercise (“Kitty Catch”) requires the user to focus their eyes on the target, then turn their head to face the target. The cat's paws then swat the target to the opposite side and the user repeats the eye and then head movement. **(C)** The remembered target exercise (“Smashy Castles”) requires the user to look at a target on the castle, they then close their eyes and turn their head while maintaining their eyes on the remembered target. When they open their eyes, the damage caused to the castle is based on the accuracy of eye position. Copyright (2024) Blue Marble Health. Reprinted with permission.

The VORx1 and VORx2 games use a “grow plants in your garden” game mechanic in which the patient selects which type of flower or plant they wish to grow, then they “grow” the plant by performing accurate head and eye movement (Figure 2A). For the Gaze Shifting exercise, the theme is “kitty catch” in which the user focuses their eyes on the target, then moves their head left and right to cast the target to the other side of the screen. The cat's paws push the target back and forth. The objects that are tossed back and forth are random (e.g., taco) and the backgrounds can be adjusted for increased complexity (Figure 2B). The Remembered Target game uses a “smashing castles” game mechanic in which the patient selects the castle (of increasing complexity) they wish to destroy (Figure 2C). They “destroy” the castle by maintaining gaze on the target after closing the eyes, turning the head and then re-opening the eyes.

Participants were asked for feedback about their experience with the APP each week, including any technical issues and the usefulness of the game feedback (e.g., cues to increase head speed or maintain eyes on target). Participants identified positive aspects and some challenges of the games. Specific recommendations for improving the APP experience included streamlining instructions to speed up play and adding additional plant options for Gaze Garden (VORx1) which only had four options that became boring after using the APP for 4 weeks.

**Figure 3:**
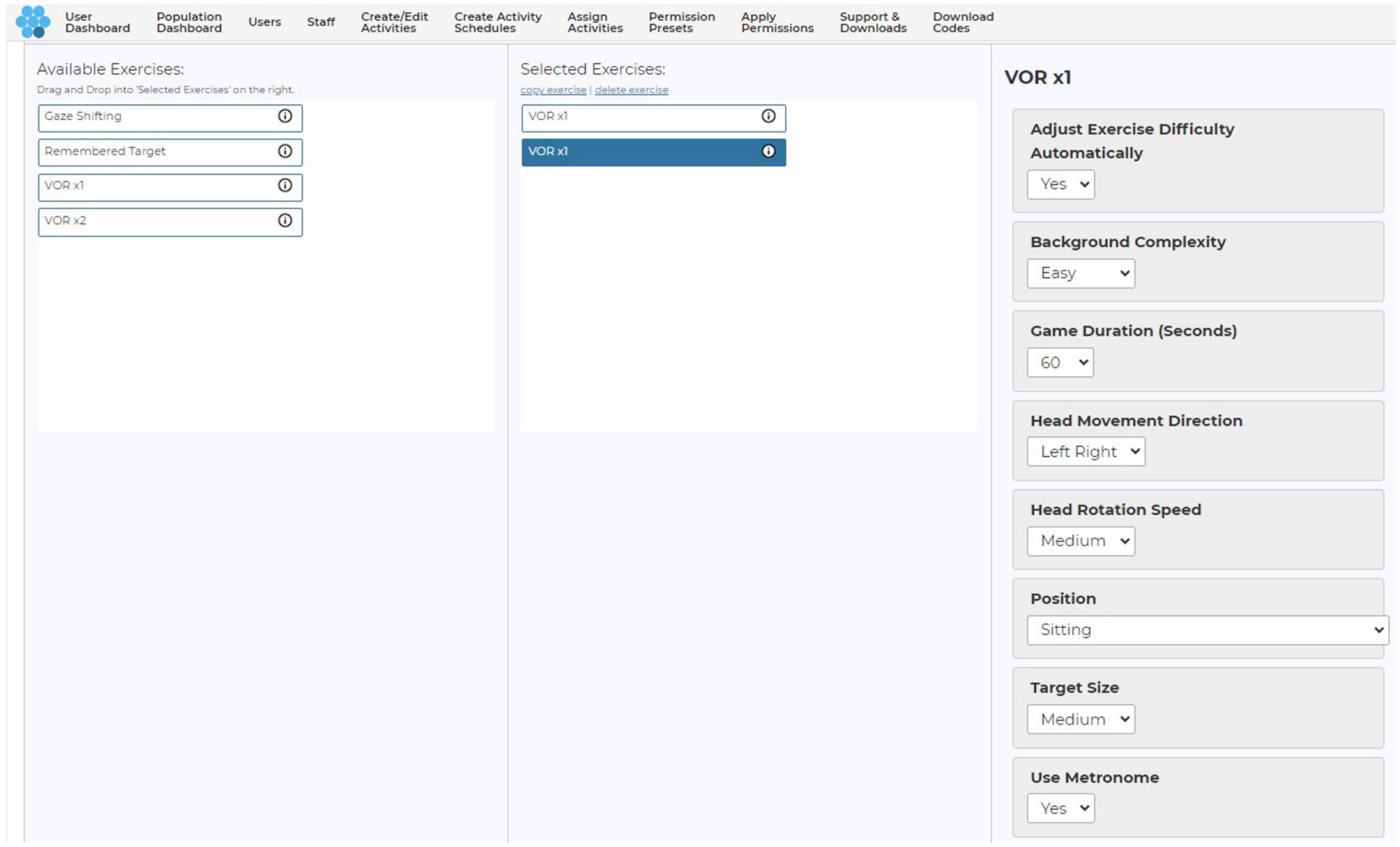
Clinician user interface and adjustable parameters. Copyright (2024) Blue Marble Health. Reprinted with permission.

Parameters of the games that could be controlled by the clinician from a web-based user-interface included manipulation of duration (30–120 s), direction of head movement (horizontal, vertical), target head speed (horizontal: slow −60°/s, medium −120°/s, fast −180°/s, and fastest −240°/s; vertical: slow −45°/s, medium −78°/s, fast −88°/s, and fastest −95°/s), target size (small, medium, and large) and background complexity (low complexity to high complexity; Figure 3). Target size is a function of the screen size (Supplementary Table 5).

#### 3.2.3 Integration of sensor

**Figure 4:**
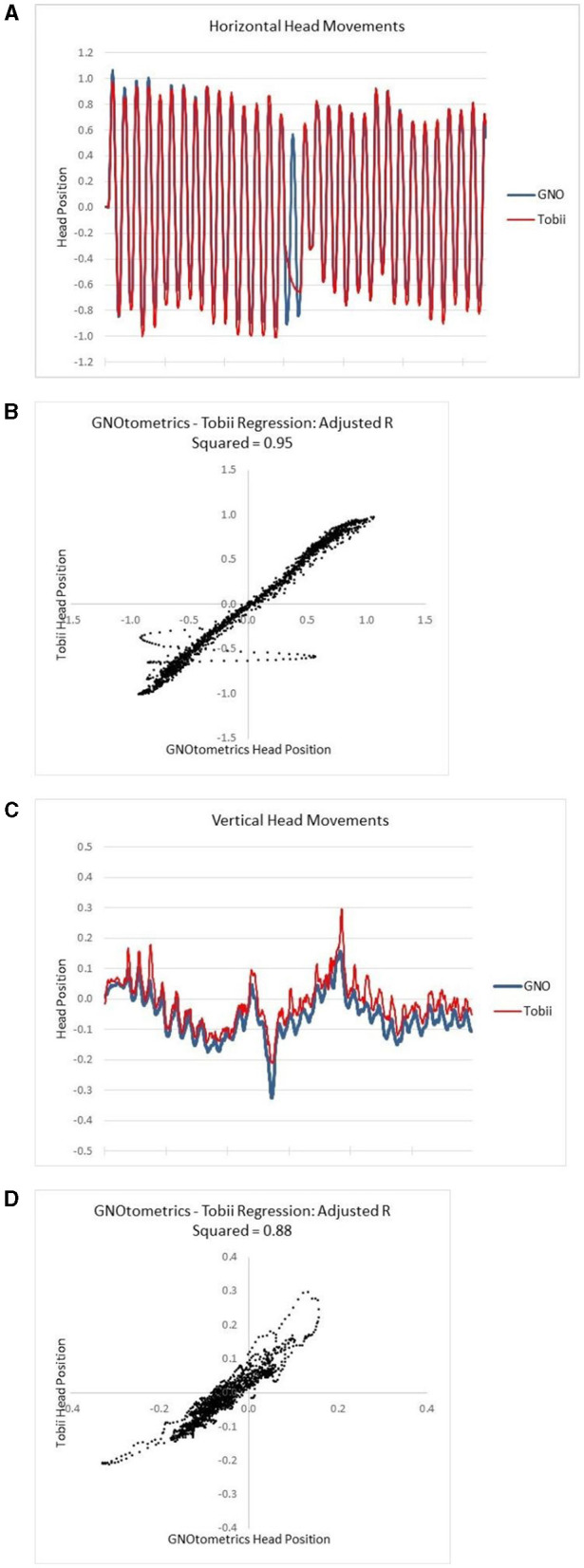
Alignment of Tobii head tracking with IR goggle angular rate sensor data. **(A)** Raw data for horizontal head movements; **(B)** the regression analysis for the horizontal movements; **(C)** raw data for vertical head movements; **(D)** the regression analysis for the vertical movements.

**Figure 5:**
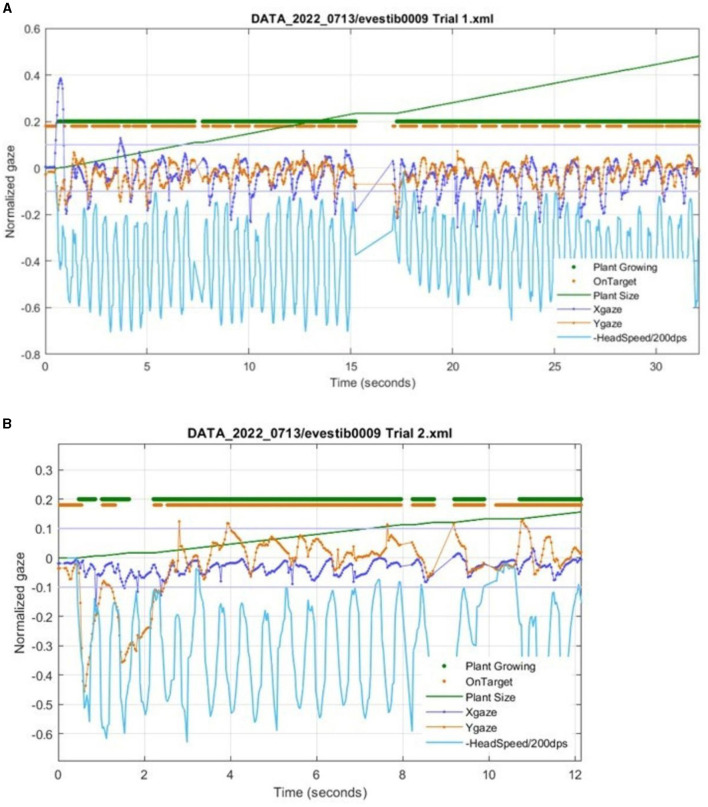
Metrics from Gaze Garden (VORx1) of a participant with unilateral vestibular hypofunction using the Tobii eye and head tracker. The data are directly from HiM-V output without processing. **(A)** The Xgaze and Ygaze lines show the normalized horizontal and vertical eye gaze deviation relative to the center of the on-screen target. The thick Plant Growing line (at an arbitrary vertical position) shows each data sample with a dot when the plant is growing. Similarly, the OnTarget line shows a dot when the subject gaze is within a −0.1 to 0.1 normalized range (shown by thin gray lines). Note that the gaze positions which exceed the +/−0.1 limit correspond to gaps in the OnTarget trace. **(B)** A zoomed-in portion from the same subject during a different trial. Note that around 2 s, Plant Growing is off, due to the eye being off the target for too long. Note that after 10 s, there is a period of low head velocity, and the plant correctly stops growing.

The Tobii eye and head tracker was found to have variable alignment with IR goggle data. The adjusted *r*^2^ values for horizontal head rotations ranged from 0.62 to 0.99 for the five subjects. There was less agreement with the vertical head movements, as we were unable to align and analyze the vertical head movements in three of the five subjects. For the two individuals where we could assess the vertical head movements, the adjusted *r*^2^ values were 0.14 and 0.88. There was some disparity in the head position measurement between the two devices at the extremes of the head movement (Figure 4). With the Tobii eye and head tracking data, the in-app visual feedback design (plant size, feedback related to head speed and gaze stability on target) provided appropriate feedback to users in terms of game metrics (Figure 5).

During head tracker validation for dDVA, the total system latency (i.e., latency between head motion tracked by Tobii and graphic display in the HiM-V platform) was found to be ~130 ms. This delay was not problematic for game play but was problematic for developing a digital DVA, where precise timing was required to display the optotype while the head was near peak velocity. To compensate for the system latency, an array of recent head position samples from the Tobii were used to anticipate 130 ms in advance of when the head position would cross the zero/straight-ahead position, which would also be where the head velocity was at or near its peak. To predict when the head was facing straight ahead, the algorithm determined if the head position was <20° from the center and moving toward the center faster than the minimum threshold in degrees/second. The pace of head movement was defined as the time to move the head in one direction (i.e., from left to right or right to left), and was identical to the metronome beat interval used to pace the participant in turning the head. For example, at a metronome pace of 60 bpm, it would take 1 s (i.e., one metronome beat) to turn the head from left to right and 1 s from right to left, equaling 30 cycles of head turns per minute or 0.5 Hz.

**Figure 6:**
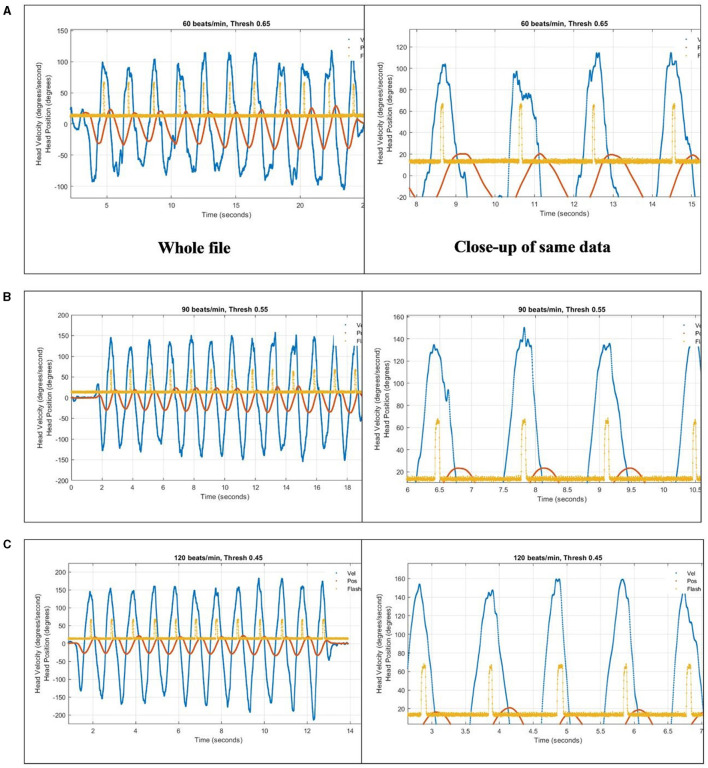
To compensate for a 133-ms reporting latency, an array of recent head position samples from the Tobii were used to anticipate head position crossing the zero/straight-ahead position, which was also where head velocity was at or near its peak. The algorithm was tested at three different head speeds paced by a metronome in the app: **(A)** 60 bpm, **(B)** 90 bpm, and **(C)** 120 bpm. Blue trace is head horizontal gyroscope velocity data. Orange trace is integrated gyroscope data, to give head position. Yellow trace is output of light sensor aimed at location of optotype on computer screen, showing when the letter turns on as an upward pulse.

The algorithm for dDVA was tested at three different head velocities (60, 90, and 120 bpm). At 60 bpm, the head excursion required was too large and uncomfortable. Also, the peak head velocities were a bit slow at ~100°/s and the appearance of the optotype occurred consistently during the period of highest head velocity, sometimes before the peak (Figure 6A). At 90 bpm the head excursion was more comfortable and the appearance of the optotype began consistently near the peaks, rarely earlier than the peak with peak head velocities consistently at or above 120°/s (Figure 6B). At 120 bpm the head excursion was comfortable although the pace was very quick. The appearance of the optotype occurred after the peak head velocity, which were consistently above 140°/s, and turned off after the head slowed (Figure 6C).

### 3.3 Pilot intervention study

#### 3.3.1 Participants

Of the 32 participants who were enrolled, 25 completed the study intervention (of these eight were not randomized to group), five withdrew and two were disqualified. Three of the participants chose to change group assignment during the 4-week intervention with two of these changing to S-VRT due to lack of confidence using technology. There were no significant differences (*p* > 0.05) among the subjects who withdrew/disqualified or the intervention groups (S-VRT and D-VRT) in terms of age, gender or cognition. The majority of participants were male (five female, 20 male) with a mean age of 66.8 ± 11.9 years (range: 28–80; Table 4).

**Table 4:** Participant demographics (*n* = 25) and adherence by group (S-VRT vs. D-VRT) for the pilot intervention study.

**Variable**	**All subjects (*n* = 25)**	**S-VRT group (*n* = 13)**	**D-VRT (*n* = 12)**
**Age (years)**			
Mean (SD)	66.8 (11.9)	65.2 (14.5)	68.5 (8.4)
Range	38–80	38–80	54–75
**Gender (** ***n*** **)**			
Female/male	5/20	3/10	2/10
**Mini-mental Status Exam**			
Mean (SD)	28.9 (1.1)	28.8 (1.1)	29.1 (1.2)
**Race (** ***n*** **)**			
White	22	11	11
Black	1	1	0
Not reported	2	1	1
**Vestibular diagnosis (** ***n*** **)**			
UVH	22	11	11
BVH	3	2	1
**Education**			
Vocational/some college	14	6	5
BA/BS	5	2	2
MA/MS	7	3	4
Professional	2	1	0
**Assistive device use**			
None	17	8	9
Cane/walking stick	4	3	1
Walker	2	1	1
**Adherence (%)**			
GSE		66.2 (17.0)	71.2 (29.6)
Balance		77.2 (20.6)	86.1 (14.8)

S-VRT, Standard vestibular rehabilitation therapy; D-VRT, Digital vestibular rehabilitation therapy; UVH, unilateral vestibular hypofunction; BVH, bilateral vestibular hypofunction; GSE, Gaze stabilization exercises; Balance, Balance and gait exercises.

#### 3.3.2 Outcome measures

There was a significant main effect of time and no significant main effect of group (*p* ≥ 0.06) or significant interaction (*p* ≥ 0.06) for ABC (*F*_1, 23_ = 14.84, *p* < 0.001, η^2^ = 0.39), DZ VAS (*F*_1, 22_ = 18.56, *p* < 0.001, η^2^ = 0.46), DVA (*F*_1, 10_ = 16.39, *p* = 0.002, η^2^ = 0.62), Gait Speed (*F*_1, 20_ = 4.57, *p* = 0.045, η^2^ = 0.18), and mCTSIB (*F*_1, 21_ = 9.36, *p* = 0.006, η^2^ = 0.31) indicating improvement following VRT (Table 5). There were significant interactions (Time by Group) for DHI (*F*_1, 23_ = 7.67, *p* = 0.01, η^2^ = 0.25) and FGA (*F*_1, 22_ = 10.17, *p* = 0.004, η^2^ = 0.32) and no significant group effects (*p* > 0.08). *Post-hoc* univariate analyses determined that there was a significant difference for DHI and FGA (*p* = 0.01, *p* = 0.009, respectively) between groups at baseline, but not at post-test (*p* ≥ 0.70). S-VRT improved significantly from baseline to post-test for DHI and FGA (*p* = 0.002, *p* < 0.001, respectively). D-VRT improved significantly from baseline to post-test for FGA (*p* < 0.001), but not for DHI (*p* = 0.12).

**Table 5:** Mean (STD) of rehabilitation outcomes after 4 weeks of VRT by group (S-VRT vs. D-VRT).

	**S-VRT (*****n*** = **13)**		**D-VRT (*****n*** = **12)**		Significance (*p*-value) of time/interaction of time x group^*^
**Measure**	**Pre**	**Post**	**Pre**	**Post**	
DHI (/100)	50.31 (22.06)	23.69 (15.12)	29.83 (13.92)	24.33 (14.11)	<0.001/0.011
ABC (%)	55.53 (23.28)	78.49 (16.75)	75.49 (16.75)	83.10 (12.51)	<0.001/0.065
DZ VAS (/10)	2.97 (2.31)	0.80 (1.01)	2.49 (2.05)	0.77 (1.50)	<0.001/0.62
DVA (LogMAR)	0.45 (0.19)	0.36 (0.19)	0.50 (0.19)	0.36 (0.15)	0.002/0.41
Gait Speed (m/s)	0.85 (0.26)	1.00 (0.21)	1.04 (0.35)	1.05 (0.23)	0.04/0.09
FGA (/24)	14.92 (5.27)	23.15 (4.69)	20.36 (3.64)	23.91 (4.81)	<0.001/0.004
mCTSIB (s)	88.83 (34.69)	111.43 (10.19)	100.88 (16.59)	112.64 (7.16)	0.006/0.37

VRT, Vestibular rehabilitation therapy; S-VRT, Standard VRT; D-VRT, Digital VRT; DHI, Dizziness Handicap Inventory; ABC, Activity-specific Balance Confidence scale; DZ VAS, Dizziness Visual Analog Scale; DVA, Dynamic Visual Acuity (of the affected side for UVH or mean of both sides for BVH); FGA, Functional Gait Assessment; mCTSIB, modified Clinical Test of Sensory Interaction and Balance.

^*^2 x 2 (Time by Group) RM ANOVA were performed and there were no significant main effects of Group (p ≥ 0.06) so individual p-values are not reported. Exact p-values for main effects of Time (pre vs. post) and significant interactions (Time by Group) are reported.

#### 3.3.3 Adherence and system usability

There were no significant group differences (*p* ≥ 0.29) in exercise adherence for either GSE or balance and gait exercises (Table 5). Both groups performed at least two-thirds of prescribed GSE and at least three-quarters of prescribed balance and gait exercises.

Eight participants completed the System Usability Scale for a mean standard score of 82.5 (range: 57.5–100) indicating excellent ease of use equivalent to an A score (13). Two of the eight participants scored the APP lower than 70 (equivalent to C and D scores) in terms of usability.

## 4 Discussion

The goal of this study was to develop and evaluate a remote therapeutic monitoring VRT Platform app for the assessment and treatment of vestibular/balance impairments that would foster adherence to prescribed HEP at the recommended intensity and dose and provide feedback for accurate performance of gaze stabilization exercises. Both participants with chronic dizziness and experienced vestibular clinicians felt that a VRT app would be beneficial for vestibular rehabilitation outcomes and provided specific features that the app should include. Commonly used PROs for vestibular assessments were integrated into the app including the mABC, DHI, DRS, VAS for Dizziness Interference, Oscillopsia, Disequilibrium, and Dizziness with head movement, and mMST. Each were determined to have excellent scores (A+ score) for usability and learnability by participants with chronic dizziness and testers. Generally, the outcome measures demonstrated good to very good validity in comparison with the standard clinical (paper and pencil) version of the measures and very good test-retest reliability. The game mechanics and control of the game by means of eye and head movements was acceptable to perform prescribed gaze stabilization exercises. Use of the HiM-V APP for the gaze stabilization exercises was feasible and outcomes relevant to gaze stabilization exercises (i.e., DVA and DZ VAS) were not different to standard VRT.

Our goal was to develop games that would be appeal to a broad audience (some of whom are veterans with PTSD) of different ages and genders. We brainstormed several game themes, some of which were unable to satisfy the unique requirements of VRT. In general, the feedback about the games and themes has been positive (“Love the garden concept!”). The primary user comments have been about how to improve the usability and efficiency of the games and how to improve communication of the game mechanics. It should be noted that the re-skinning of the games is relatively easy compared with developing an effective game mechanic. Thus, at this stage of development we prioritized ensuring that the games guided the patient in correct performance of the movement.

Because our VRT Platform does not incorporate a virtual reality head mounted display or a wide field of view (FOV), it is not immersive like high end virtual reality systems and unlikely to induce simulator sickness (25). We did not receive any reports of motion sensitivity relative to using the gamified VRT Platform.

### 4.1 Patient perspective

The prevalence of vestibular disorders increases with age; thus, it is important to consider the needs and preferences of older adults in designing technology based VRT. Participants with chronic dizziness (>50 years old) in the current study reported regular use of technology and 50% had prior experience with VRT. Overall, the participants with dizziness in the current study (mean age = 66.8 years, range: 38–80 years) rated the HiM-V APP very highly (SUS score of 82.5 = A) for usability and learnability. Many of these participants felt that the traditional home exercise programs were not easy to adhere to and that forgetting was a barrier to exercise adherence. Participants with dizziness reported various solutions to these barriers that included the use of digital reminders, comments about the APP being engaging, and supported feedback in the form of a summary report to be shared with their clinician. Participants also provided feedback for suggested improvements including using a more streamlined interface, using more adult themes, and including more variety in the gaming. Participants also enjoyed the competitive aspect of the games, liked the on-screen feedback indicating whether they were doing the exercises correctly and commented that use of a metronome for pacing head movement was novel. Participants particularly appreciated that two of the games provided a score at the end which provided feedback related to performance. In contrast, the VORx1 and VORx2 games (Gaze Garden) did not provide a score and participants reported being unsure as to how the size of the plant growth related to the accuracy of their performance and as such, they did not know how well they had performed. Some participants reported technical difficulties, especially with calibrating the Tobii eye and head tracker, which reduced enthusiasm for the APP.

Multiple studies have reported that older adults find technology-based exercise programs to be enjoyable, acceptable, and motivating (26). D'Silva et al. reported that their older female participants (aged 60–74 years) found that the Vestibular Rehabilitation App^TM^ was easy to learn in a single session, the game format was enjoyable due to the graphics and colors, the score was motivating, and the game feedback would improve performance (27). Likewise, Meldrum et al. reported that participants with dizziness (mean age = 59 years, range: 38–76 years) found a digital VRT app to be easy to use (SUS = 82.7 = A), useful in learning about their vestibular condition, and enjoyable due to the color scheme and clear layout (28). Developers of technology based VRT, do need to consider adult preferences in their game design. One web-based VRT system was found to have good usability (mean SUS score of 77.8 = B+) but received criticism for lack of interaction and being visually unattractive (29).

### 4.2 Clinician perspective

Experienced VRT clinician participants felt that a VRT app should include features to record and track head/eye movement, monitor symptoms, score the accuracy of task performance, and measure and improve adherence to a prescribed home exercise program. Despite clinicians' desire to be able to track both head and eye movements, to date most VRT apps only monitor head movement (27, 28); thus, the ability to assess accurate performance of gaze stabilization exercises is limited. To the best of our knowledge, VestAid is the only other VRT system in development to monitor eye movement (30).

The HiM-V app is intended to be utilized as an adjunct to VRT and not without the guidance of a vestibular clinician. The features requested by experienced vestibular clinicians in the current study are consistent with those requested in other studies (27, 28). The HiM-V APP includes VOR exercises prescribed as part of a gaze stabilization exercise program, and include the VORx1, VORx2, gaze shifting, and remembered target exercises in addition to commonly used PROs. D'Silva et al. interviewed experienced clinicians who identified VORx1 (pitch/yaw) and gaze shifting exercises as the primary gaze stabilization exercises prescribed (27). The ability to monitor exercise adherence and accurate exercise performance as well as symptom provocation were considered important attributes of the HiM-V app. The web-based clinician interface allows the therapist to remotely monitor patient performance and adjust exercise parameters as necessary. Currently clinical practice forces clinicians to rely on patient's self-reported compliance using a paper exercise log or patient memory of exercise performance, both of which are likely to be inaccurate. Clinicians in the D'Silva study wanted the ability to prescribe exercise duration, to measure symptoms of dizziness following exercise performance and improve exercise adherence (27). To improve adherence the app includes automated reminders, a game format, and game rewards including trophies and scores.

### 4.3 Integration of PROs in HiM-V

Commonly used PROs for vestibular assessments (e.g., Activities-specific Balance Confidence Scale, Dizziness Handicap Inventory, and Disability Rating Scale) were successfully integrated into the APP. In general, the PROs were valid and reliable compared to standard paper and pencil versions, and the usability scores were excellent (A+) suggesting that patients could easily follow the instructions to complete the questionnaires on the app. Administration of PROs via an app saves time for the clinician by eliminating scoring and interpretation, and also enables the remote monitoring of any changes in symptoms. The PROs included in the HiM-V app are consistent with those featured in other systems (27, 28).

### 4.4 Integration of eye and head sensor into app

Adequate head speed and accurate eye movement is linked to rehabilitation outcomes, yet no commercially available home program has the capability to provide clinicians with head speed and accuracy of eye movement performance data to correct improper performance. A primary goal of the study was to develop a remote therapeutic monitoring VRT Platform APP that would provide this feedback for accurate performance of gaze stabilization exercises. The Tobii eye and head tracker, designed for online gaming and not for health-related interventions, was chosen because it is the only off-the-shelf sensor that tracks both eye and head movement, which together provides users with a measures of gaze position (requires knowing eye and head position). The Tobii eye and head tracker was an acceptable method for tracking compliance of gaze stability exercises for the desired head speed and gaze stability accuracy but was inadequate for use in a digital DVA test.

The data comparing the output of the HiM-V plus the sensor system to that from the GNOtometrics system suggest that the system is capable of accurately measuring head position. However, this was not a universal finding in the subjects used in the validation portion of the study. When the system worked, it worked very well with adjusted *r*^2^ values up to 0.99 for horizontal head movements (*r*^2^ range: 0.62–0.99; mean: 0.89). Vertical head movements were problematic as we were unable to match up the sensor output and GNOtometrics data for three of the five subjects. For the two subjects we were able to analyze vertical head movement data, the *r*^2^ values (0.14 and 0.88) were lower than those for the corresponding horizontal head movements (0.99 and 0.95, respectively). There is some discrepancy in the output from the two systems at the extremes of the head movement (Figure 4). At the extremes of the head movement, the head velocity is at the lowest point, and goes to 0°/s; as such, this portion of the head movement is not critical for the gaming activities.

Our data suggest that the combination of the HiM-V plus sensor can be valid for functions other than DVA testing, including ability to track head/gaze movement, monitor symptoms, score the accuracy of task performance, and measure adherence to the prescribed home exercise program. In time, we expect the sampling rates will increase, enabling creation of a valid digital DVA test. Alternatively, a different method to track gaze and head movement may be an option. Hovareshti et al. used the video captured by a tablet to estimate head angles and determine eye-gaze compliance (30). They assessed the accuracy of the system and found time errors <100 ms and mean head angle errors <10° that may be adequate for therapist to assess patient performance of gaze stabilization exercises. With the democratization of artificial intelligence and machine learning, more advanced machine learning algorithms may estimate gaze and head movement more accurately and could be explored in future iterations.

### 4.5 Feasibility study

The findings of the current study were in alignment with the published evidence for VRT and provide initial evidence in support of a digital VRT APP with gamified gaze stabilization exercises reducing symptoms of dizziness and imbalance and improving postural and gaze stability (4). A larger scale feasibility study would be a next logical step including outcomes such as safety, adverse events, effect size, cost-utility, and engagement. Participants improved significantly following VRT for each of the outcome measures, including DVA a functional measure of the VOR. The only significant Time by Group interactions that demonstrated that the D-VRT did not improve following VRT was for DHI. The lack of improvement in DHI is puzzling but may be related to their perceived disability being relatively low on average (in comparison to the S-VRT group) at the beginning of the intervention. An important goal of creating a game format for gaze stabilization exercises was to improve exercise adherence. Adherence to gaze stabilization exercises has been reported to be modest at best [60%; (11)] and recently a digital VRT app reported adherence of 30% (28). Our study compared exercise adherence between groups using paper exercise diaries but did not find a difference (66% for S-VRT and 71% for D-VRT). It is unclear why adherence in the present study was so high compared to Meldrum et al.; although, weekly supervised visits (either in-person or via phone call) and participant motivation may have contributed to the findings. The study sample in the current study is small so further study is warranted. The majority of participants remained in the group to which they were randomized with only two participants requesting to leave the APP group due to issues with technology. It may be helpful in future studies to identify people most likely to benefit from a technology-based VRT approach.

### 4.6 Limitations

The pilot study had a small sample size that was predominantly male and with chronic symptoms, thus we cannot generalize our findings to adults with acute onset of symptoms. There is the potential that the higher percentage of males in our sample may have introduced bias in terms of confidence with using technology. However, we feel that this effect would be minimal based on the findings of Meldrum et al. of no gender difference in system usability scores of the Wii Fit system to train balance (31). Inclusion of a more diverse sample of participants that are more representative of the population with vestibular dysfunction in a subsequent study would more strongly support feasibility of D-VRT. The majority of participants remained in the group to which they were randomized with only two participants requesting to leave the APP group due to issues with technology. It may be helpful in future studies a priori to identify those patients most likely to benefit from a technology based VRT approach. Secondly, we used a paper exercise log to compare adherence between groups; however, exercise diaries may be inaccurate. Finally, we were unable to develop a reasonable digital DVA due to the inability to capture eye and head movements given the sensor's capabilities and intended use for non-health related gaming purposes.

## 5 Conclusions

Adults with chronic dizziness and vestibular therapists are receptive to the use of technology for VRT and the HiM-V Platform was found to be feasible in adults with chronic peripheral vestibular hypofunction. Future iterations of HiM-V should incorporate patient and clinician feedback to optimize their experience. The vestibular clinical guidelines strongly recommend supervised VRT and the HiM-V Platform is in alignment with the guidelines by allowing clinicians to remotely monitor patient progress and remotely adjust exercises as needed. HiM-V Platform may be a useful adjunct to promote exercise adherence to gaze stabilization exercises.

## Data availability statement

The raw data from the feasibility study supporting the conclusions of this article will be made available by the authors, without undue reservation.

## Ethics statement

The studies involving humans were approved by the East Tennessee State University/James H. Quillen VA Medical Center (JHQVAMC) Institutional Review Board (IRB #0417.3s), the VA Central Institutional Review Board (Protocol #1717092), and the Alpha Institutional Review Board as the Central IRB for four additional sites (IRB #2020.1-0201). The studies were conducted in accordance with the local legislation and institutional requirements. The participants provided their written informed consent to participate in this study.

## Author contributions

CH: Conceptualization, Data curation, Formal analysis, Funding acquisition, Methodology, Resources, Validation, Writing – original draft, Writing – review & editing. SF: Conceptualization, Funding acquisition, Methodology, Project administration, Validation, Writing – review & editing. RC: Formal analysis, Methodology, Validation, Writing – review & editing. DR: Methodology, Validation, Visualization, Writing – review & editing. KS: Data curation, Methodology, Validation, Writing – review & editing. WP: Methodology, Validation, Writing – review & editing. DM: Software, Validation, Writing – review & editing. MS: Data curation, Methodology, Validation, Writing – review & editing.
