# Supplementary material for: Remote assessment and management of patients with dizziness: development, validation, and feasibility of a gamified vestibular rehabilitation therapy platform
Source: Front Neurol. 2024 May 30;15:1367582. doi: 10.3389/fneur.2024.1367582 (PMC11169667; doi:10.3389/fneur.2024.1367582)
Supplement: Supplementary file 1 [file Table_1.DOCX]

Supplementary Material

**Supplementary Table 1.** Demographic and descriptive data of participants with chronic dizziness who completed semi-structured interviews

| Age (years) | 69.6 ± 5.8  Range: 53 - 76 |  |
| --- | --- | --- |
| Education (n) | ≤ High school/GED | 4 |
|  | Some college/AA | 3 |
|  | Bachelor’s degree or higher | 7 |
| History of Falls (n) | Yes | 10 |
|  | No | 4 |
| Vestibular Diagnosis (n) | Unilateral vestibular hypofunction | 8 |
|  | Bilateral vestibular hypofunction | 2 |
|  | Benign paroxysmal positional vertigo | 1 |
|  | Normal vestibular function/ imbalance | 3 |

**Supplementary Table 2.** Technology use endorsed by participants with chronic dizziness

| Technology Use | | |
| --- | --- | --- |
| Most Frequently Used (n) | Phone | 12 |
|  | Computer | 10 |
|  | Tablet | 4 |
|  | Cochlear implant or hearing aid | 2 |
|  | TV | 2 |
| Type of Computer Owned or Used (n) | Desktop and/or laptop | 12 |
|  | Do not use a computer | 2 |
| Type of Tablet (n) | Kindle | 2 |
|  | Acer | 1 |
|  | Apple | 1 |
|  | Dell | 1 |
|  | Samsung | 1 |
|  | Not sure | 1 |
|  | Do not own | 7 |
| Top Uses of computer/tablet (n) | Search Engines | 10 |
|  | Email | 9 |
|  | Shopping | 5 |
| Comfort Level with technology (n) | Yes | 12 |
|  | No | 1 |
|  | Somewhat | 1 |
| Interaction Method | Mouse/keyboard only | 8 |
|  | Touch screen only | 1 |
|  | Mouse/keyboard AND touch screen | 4 |
|  | Does not use computers | 2 |
| Tech-savvy | Yes | 3 |
|  | No | 9 |
|  | Somewhat | 2 |

**Supplementary Table 3.** Concepts regarding program adherence from participants with chronic dizziness

| **Concept** | **Initial Coding** | **Interpretative Summary** |
| --- | --- | --- |
| Barriers to Program Adherence | "forgetting"  “requires a lot of energy”  "forgot, hard to incorporate"  "did not help"  "not all easy"  "would benefit"  "aggravating"  "get back to normal again"  "Thought it would help"  "easy to use & understand results" | **PROGRAM** **ADHERENCE** will rely on 1) alleviating/ improving symptoms 2) simplistic design and operation ease of the proposed technology |
| Sticky Interface | "feedback, reminders, ability to send info to MD"  "if it told what to do"  "If it had an alarm"  "communicate with provider"  "assess my status"  "give feedback"  "graph to see"  "reminder to do exercise"  "did not forget once in routine"  "voice to tell"  "show progress" | **STICKY INTERFACE** connects the ‘must have’ needs to create long- standing use of the proposed technology |

**Supplementary Table 4.** Concepts regarding app development endorsed by experienced vestibular clinicians/researchers

|  | **Supporting Codes** | **Interpretive Summary** |
| --- | --- | --- |
| *PERFORMING TASKS* | “weekly report would allow us to study dose” “get the best score and then try to improve the score next time”  “compile outcomes by diagnosis (e.g., neuritis vs other diagnoses) then could compare outcomes”  “raw data and then weekly summaries”  “aggregate data”  “show improvement at least monthly”  “head movement induced dizziness” | PERFORMING TASKS would report the exercise and questionnaire results back to the clinician & MD for evaluation and possible modification of treatment. |
| ADHERING TO THE PROGRAM | “gain adherence if personal interest, rewards”  “get better compliance if a game”  “help compliance”  “at home with own device”  “Step by step goal achievement”  “Are they doing the exercise?”  “duration of exercise”  “adherence (frequency per day)”  “Games would increase motivation”  “Record the # times patient did exercise and can verify adherence (frequency and how ‘well’ they did exercise)” | ADHERING TO THE PROGRAM emphasizes the need to focus on patient adherence to measure their process outside of the clinic setting to help decrease symptoms and increase patient success. |
| TRACKING MOVEMENT | “head movement excursion, velocity, and maybe acceleration”  “Head vs. eye”  “kinematics of head movement to look for avoidance”  “head speed”  “#head turns/minute”  “Gaze stability”  “eye/head movement” | TRACKING MOVEMENT will allow the clinician to see progress over time and track connections between movement and symptoms. |
| SYMPTOMATIC RESPONSES | “head motion-provoked symptoms”  “score symptoms”  “head movement induced dizziness”  “Symptom severity after each exercise”  “tracing of sway”  “eye movement recording”  “excursion/direction of head”  “maintain head velocity over time” | SYMPTOMATIC RESPONSES enable a clinician to monitor and identify potential triggers/movements that may cause dizziness or discomfort. |

**Supplementary Table 5.** Calculation of target size as a function of game and screen size

|  | VORx1, VOR/2 | Gaze Shifting |
| --- | --- | --- |
| Small target | Screen height/8 | Screen height /18 |
| Medium target | Screen height /6 | Screen height /14 |
| Large target | Screen height /4 | Screen height /10 |

## Supplementary Figures

**Supplementary Figure 1.** Word cloud produced from the expert clinician/researcher data and frequencies from concepts with the top 50 words. The size of each word indicates the frequencies: larger words have higher frequencies.

*
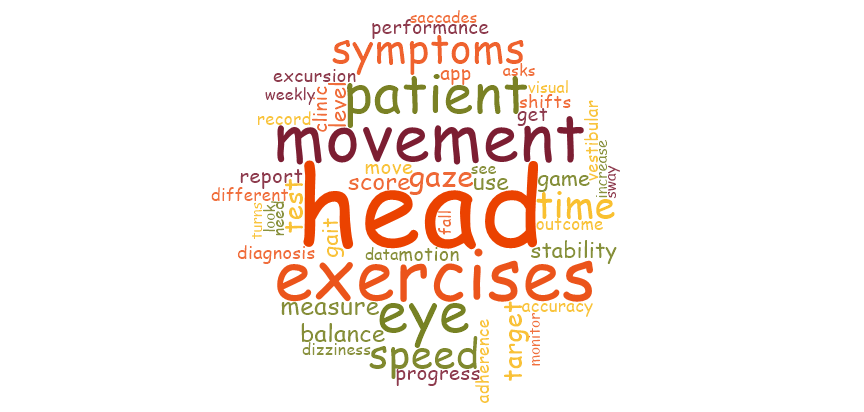
*
